# The diagnostic value of ultrasound in pediatric testicular torsion with preserved flow

**DOI:** 10.3389/fped.2022.1001958

**Published:** 2022-09-28

**Authors:** Zhihua Xu, Junbo Wu, Shuangshuang Ni, Hongxia Luo

**Affiliations:** ^1^Department of Ultrasonic Diagnosis, The Second Affiliated Hospital and Yuying Children's Hospital of Wenzhou Medical University, Wenzhou, China; ^2^Department of Children's Ultrasound Imaging, Wuhan Children's Hospital, Tongji Medical College, Huazhong University of Science and Technology, Wuhan, China

**Keywords:** testicular torsion, ultrasound, pediatric, Color Doppler, whirlpool sign

## Abstract

**Background:**

Testicular torsion is the reduction of blood flow to the testis after spermatic cord torsion. For patients, the diagnosis of testicular torsion is controversial and complicated by the fact that ultrasound blood flow signals are not significantly reduced in comparison to the unaffected, healthy, testis, despite persistent symptoms on the affected side. Our study aims to investigate the diagnostic characteristics of high-resolution ultrasonography (US) in pediatric testicular torsion with the preserved flow to increase diagnostic accuracy.

**Methods:**

Seven pediatric patients aged 49 days to 15 years old, with the preserved blood flow, but surgically diagnosed as testicular torsion, from October 2017 to August 2019, were retrospectively included in the study. The imaging manifestations of high-frequency ultrasonography were evaluated.

**Results:**

All cases had preserved testicular blood flow, but the surgical findings showed various degrees of twist, from 90 to 540 degrees. Preoperative ultrasound showed spermatic cord distortion in all cases, and testicular long axis tilting in four cases (4/7 = 57.1%).

**Conclusion:**

In some testicular torsion cases, Color Doppler may show normal or increased blood flow signals in the testis. We should further observe the morphology and position of the testes and epididymides, the echo of the testicular parenchyma, and, especially evaluate the “whirlpool sign” in the spermatic cord, to avoid missing testicular torsion with blood flow signals.

## Key points

-***What is known about the subject:*** (1) Testicular torsion is a common scrotal emergency, occurring in pediatric patients. (2) Testicular torsion is the reduction of blood flow after spermatic cord torsion.
-***What this study adds:*** (1) Preserved intratesticular blood flow may lead to a false negative diagnosis. (2) The “whirlpool sign,” heterogeneous echotexture, appearances of the epididymis are the critical features in patients with the preserved flow.

## Background

Testicular torsion is a common scrotal emergency, occurring in 5–25% of pediatric patients (1–3). The first peak onset appears within 1 year after birth, followed by a second surge in early adolescence; the incidence is 65% in the 12–18 years age cohort, due to a rapid increase of testicular volume during puberty (4). Intravaginal testicular torsion is the most common type, and it primarily occurs between 3 and 20 years of age (5, 6).

Testicular torsion is the rotation of the testis with torsion of the spermatic cord. This leads to vascular insult, which results in testicular ischemia and ultimately necrosis. To prevent testicular necrosis, an accurate and quick diagnosis is essential to determine the subsequent treatment (7). The golden time for torsion reduction is 6–8 h. The salvage rate is almost 100%, if treated within 6 h after onset, but decreases quickly with time, declining to between 80 and 88% at 12 h, 31% at 24 h, and only 2.6% at 48 h (8).

The salvage rate is < 10% if revascularization is surgically corrected after 24 h in patients with testicular torsion (9–11). Therefore, early identification of risk factors for predicting testicular torsion and potential necrosis is very important in improving children's outcomes.

A testicular ultrasonography (US) is an ideal imaging tool for diagnosing testicular torsion before attempting surgical correction. Using Color Doppler, the vascularity of the testis can be evaluated. The reduction or absence of affected testicular flow has a high diagnostic accuracy with a sensitivity of 86–100%, and a specificity of 97.9–100% (12–14). Color Doppler ultrasonography has proven particularly useful in the differential diagnosis (13, 15). However, preserved vascularity on Color Doppler does not exclude testicular torsion. Testicular torsion may present as hyperemia in its early stages due to venous dilation and preserved arterial flow (16, 17). Thus, normal or increased blood flow in the affected testis is an obstacle to the diagnosis of testicular torsion, as a result, testicular necrosis and atrophy are caused. In addition, Martha's study showed that the diagnosis of testicular torsion is complicated by the fact that ultrasound blood flow is not significantly reduced in comparison with the unaffected, healthy, testis, despite persistent symptoms on the affected side (18).

So, the preserved intratesticular flow may lead to a false negative diagnosis (6, 19). The purpose of this study was to investigate the diagnostic characteristics of high-resolution ultrasonography in pediatric testicular torsion with preserved flow, to increase the diagnostic accuracy of testicular torsion.

## Methods

### Patient cohort

**Figure 1:**
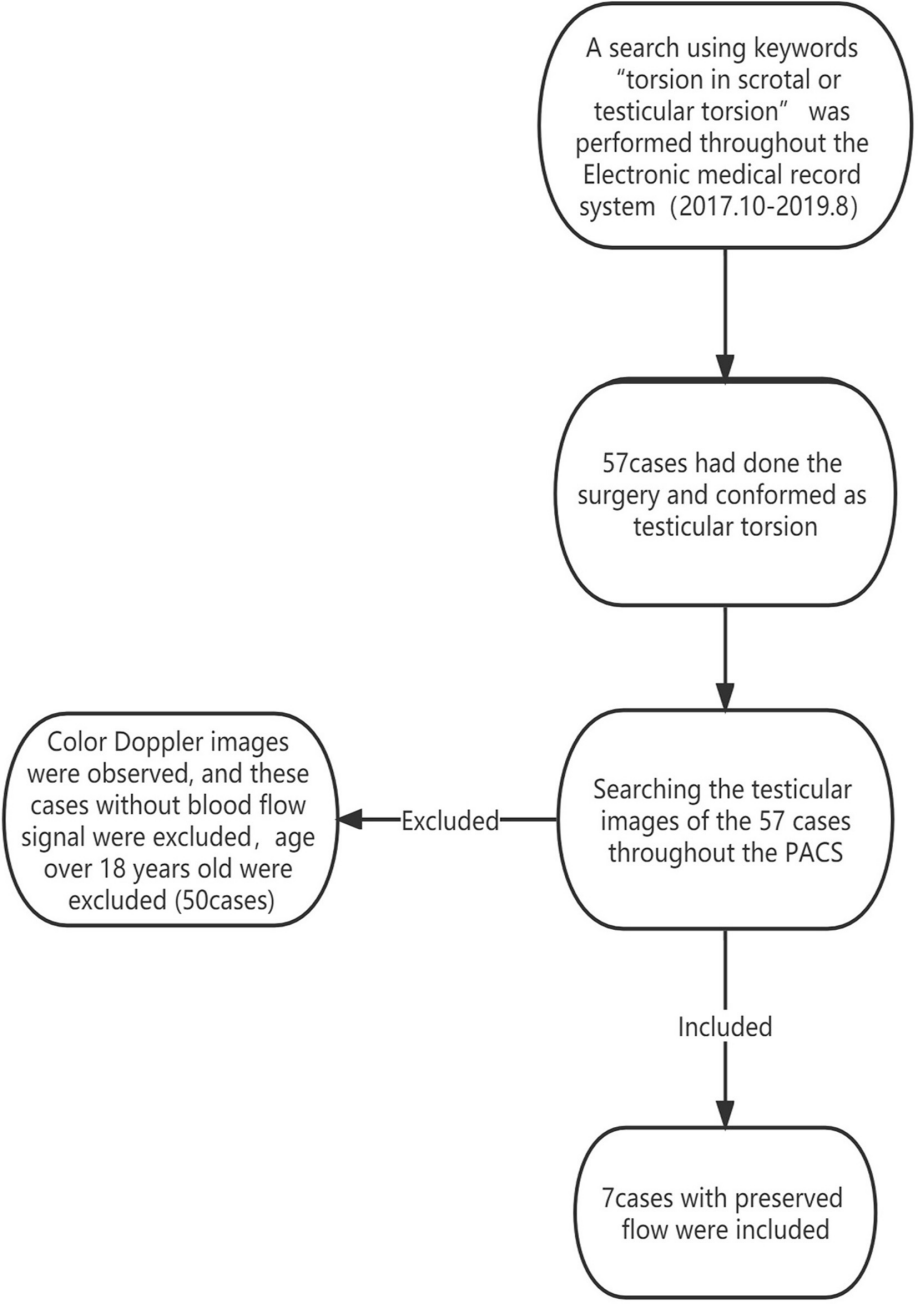
A flowchart of enrollment.

A search using the keywords “torsion in scrotal or testicular torsion” was performed throughout the Electronic medical record system from October 2017 to August 2019, to retrieve patients with testicular torsion, who were confirmed by the surgery. Then, searching these testicular images throughout the Picture Archiving and Communication System (PACS), patients with an absence of blood flow to the testis and age over 18 years old were excluded from this study. In addition, testes with the preserved flow were included (Figure 1). We obtained the surgical records of each patient from the electronic medical record system, obtained the surgical results, and verified the surgical results with the surgeon. This study was approved by the local Research Ethics Committee (Ethic Reference Number: L-2020-19); and the requirement of patient consent was waived.

### Patient and public involvement

This is a retrospective study and no patient was involved.

### Instruments and methods

All sonograms were obtained with a 5–12 MHz linear transducer (LOGIQ 7 of GE, EPIQ7 of PHILIPS). The children were placed in the supine position, and the testes were evaluated in at least 2 planes: longitudinal and transverse. Transverse images were obtained in the superior, mid, and inferior portions of the testes. Longitudinal views were obtained centrally as well as medially and laterally. Each testis was evaluated in its entirety. All the examinations were performed according to the AIUM Practice Guideline for the Performance of Scrotal Ultrasound Examinations (20).

We collected the position, size, morphology, internal echo, and the spermatic cord in the testis, epididymis, and testicular sheath cavity of pediatric patients. The size, echogenicity, and blood flow of each testis and epididymis were compared to the contralateral side. A comparison of the testes was accomplished with a side-by-side transverse image. Color Doppler sonography included at least 1 side-by-side image comparing both testes (20). Torsion of the spermatic cord was diagnosed by ultrasound whenever a degree of torsion larger or equal to one-quarter twist (90 degrees) was observed (19, 21).

### Statistical analysis

The statistical method was descriptive due to the small sample size. We used Excel for data description. We reviewed and analyzed case data on age, gender, and clinical manifestations. US findings, such as data on the position, size, morphology, internal echo, and the spermatic cord in the testes, epididymis, and testicular sheath cavity, were collected.

## Results

### Clinical manifestations

In total, fifty seven pediatric ultrasound images with testicular torsion were identified from the PACS. Of these, 50 cases without detectable blood flow were excluded. Therefore, seven cases were included in the final study. All seven pediatric patients received surgery after US examination within 3 h and were surgically confirmed to have testicular torsion.

The age of the patients ranged from 49 days to 15 years old. In addition, six patients (6/7 = 85.7%) had scrotal pain and swelling, and one patient (1/7 = 14.3%) who was 49 days old, had scrotal swellings, four (4/7 = 57.1%) on the left side and three (3/7 = 42.8%) on the right side. None of the patients reported a medical history of previous trauma. The demographics and clinical characteristics are summarized in Table 1.

**Table 1:** The demographics, clinical characteristics, ultrasound performances and matching surgical results summary.

**Sonographic findings**												**Surgical results**	
**Case**	**Age**	**Time of onset**	**Affected side**	**Size**	**Location**	**Morphology**	**Echo**	**Blood flow**	**Epididymis**	**Hydrocele**	**Spermatic cord**	**Intraoperative observations**	**The endpoint of the surgery**
1	14 years 3 months	7 h	right	increase	normal	plump	uniform	normal	swelling	yes	whirl sign	Twist 180 degrees clockwise, ruddy testicles	Orchiopexy
2	14 years 7 months	3h	right	increase	normal	plump	non-uniform	normal	swelling	yes	whirl sign	Twist 360 degrees counterclockwise, ruddy testicles	Orchiopexy
3	8 years 3months	5days	left	increase	tilt	broad bean shape	uniform	increase	swelling	yes	whirl sign	Twist 90 degrees counterclockwise, ruddy testicles	Orchiopexy
4	13 years 8 months	4days	left	increase	normal	plump	non-uniform	normal	normal	none	whirl sign	Twist 180 degrees counterclockwise, testis and epididymis were dark	Orchiopexy
5	8 years 5months	15h	left	increase	tilt	plump	non-uniform	increase	swelling	none	whirl sign	Twist 540 degrees clockwise, testicular silting purple	Orchiopexy
6	49days	4days	right	increase	tilt	broad bean shape	non-uniform	normal	swelling	yes	whirl sign	Twist 90 degrees counterclockwise, testicular silting purple	Orchiopexy
7	6 years 4months	2days	left	increase	tilt	plump	non-uniform	increase	swelling	yes	whirl sign	Twist 540 degrees clockwise, ruddy testicles	Orchiopexy

### Ultrasound performance

The diseased testicles were enlarged. Furthermore, two of seven (2/7 = 28.6%) cases were inboard bean-shaped, four (4/7 = 57.1%) cases were tilted, and five (5/7 = 71.4%) cases had heterogeneous signals of the parenchyma. Fissure-free echogenic areas were seen around the mediastinum of the testis. Color Doppler showed that the blood flow of the testis parenchyma increased in three cases (3/7 = 42.8%) compared with the opposite side, and no significant difference was found in four cases (4/7 = 57.1%). Additionally, six patients (6/7 = 85.7%) had enlarged epididymis heads and increased blood flow. Testicular hydrocele existed in five cases (5/7 = 71.4%). The spermatic cords were twisted and presented as a “whirlpool sign” in all patients (7/7 = 100%), and the boundary with the epididymis was unclear, forming pseudotumor-like nodules beneath them. The overall results are displayed in Table 1.

### Surgical results

All seven cases had unilateral testicular torsion, and all were intrathecal testicular torsions. Furthermore, two cases (2/7 = 28.6%) had a one-quarter twist (90 degrees), two cases (2/7 = 28.6%) had a two-quarter twist (180 degrees), one case (1/7 = 14.3%) had 360 degrees in torsion; and two cases (2/7 = 28.6%) were twisted at 540 degrees. The blood supply was preserved in four testicles (4/7 = 57.1%), and three cases (3/7 = 42.8%) had reversible ischemia. All 7 pediatrics were ended in orchiopexy. The matching surgical results are displayed in Table 1.

## Discussion

Testicular torsion is a time-dependent diagnosis, a true urologic emergency, and early evaluation can assist in urologic intervention to prevent testicular loss. Ultrasound is the ideal imaging modality to evaluate the scrotal contents (22–24). As the testicle twists around the spermatic cord, venous blood flow is cut off, leading to venous congestion and ischemia of the testicle. The testicle will become tender, swollen, and possibly erythematous. As the testicle further twists, the arterial blood supply is cut off, which leads to further testicular ischemia and eventually necrosis (25).

In the present study, we investigated the ultrasonographic manifestations of testicular torsion with the preserved flow in 7 pediatric patients. The results indicated that the blood flow of the testis parenchyma was not absent, and increased in three cases (3/7 = 42.8%) compared with the opposite side. The present study provided ultrasonographic manifestations of testicular torsion with the preserved flow and indicated the clinicians focus on testicular torsion with preserved for early diagnosis.

### Volume of testicle increases

**Figure 2:**
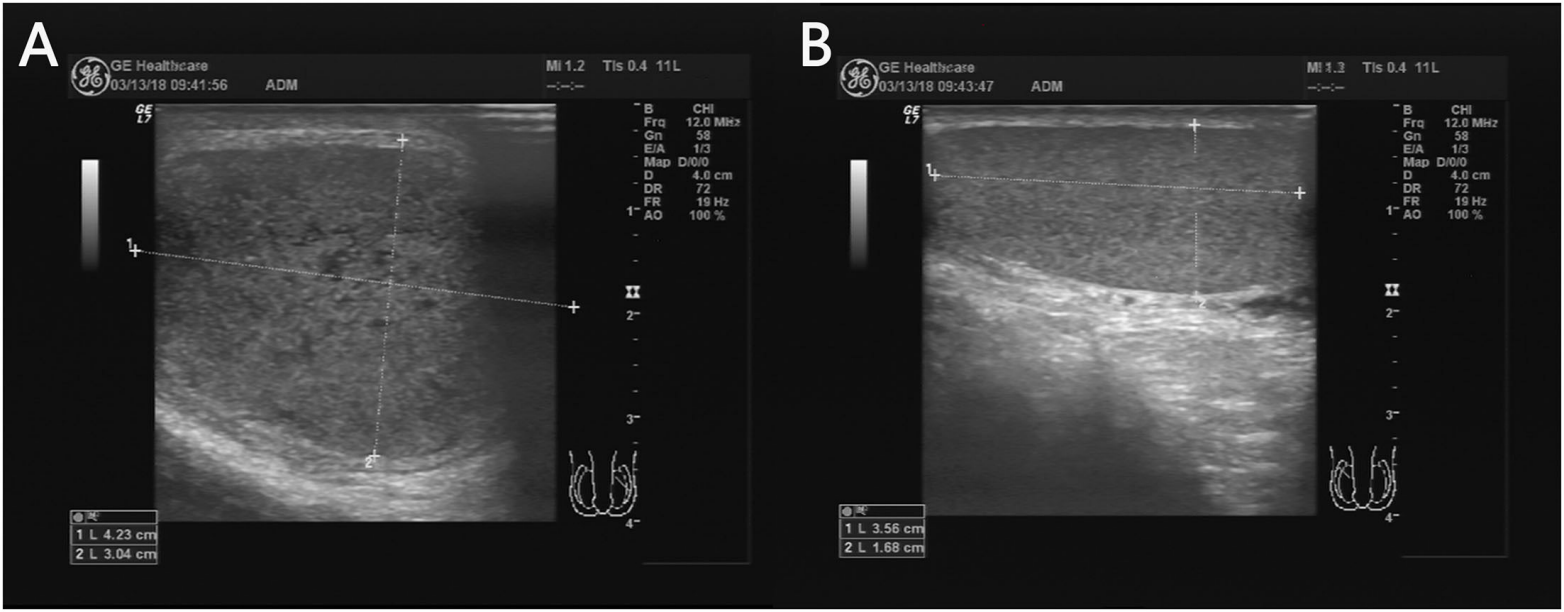
A 13 years 8-month-old boy with a first episode of acute left scrotal pain for 4 days. The left testicle is twisted **(A)** and the testicular size is significantly larger than that on the right **(B)**.

When twisted, testicles increase in size due to congestion in the parenchyma, presenting a full and spherical shape, which is often misdiagnosed as orchitis. Testicular volumes are easier for comparison to the other side. A difference in the size of the testicles on both sides, with the affected testis larger in volume than the asymptomatic side, is a crucial feature suggestive of testicular torsion (26), which is similar to the torsion of ovarian. In the present study, testicular volume on the affected side was larger than that on the contralateral side in all patients (7/7 = 100%) (Figure 2). A prospective study showed that testicular volume increased in 11 of 12 (91.7%) patients with testicular torsion (27).

### Testicular position

**Figure 3:**
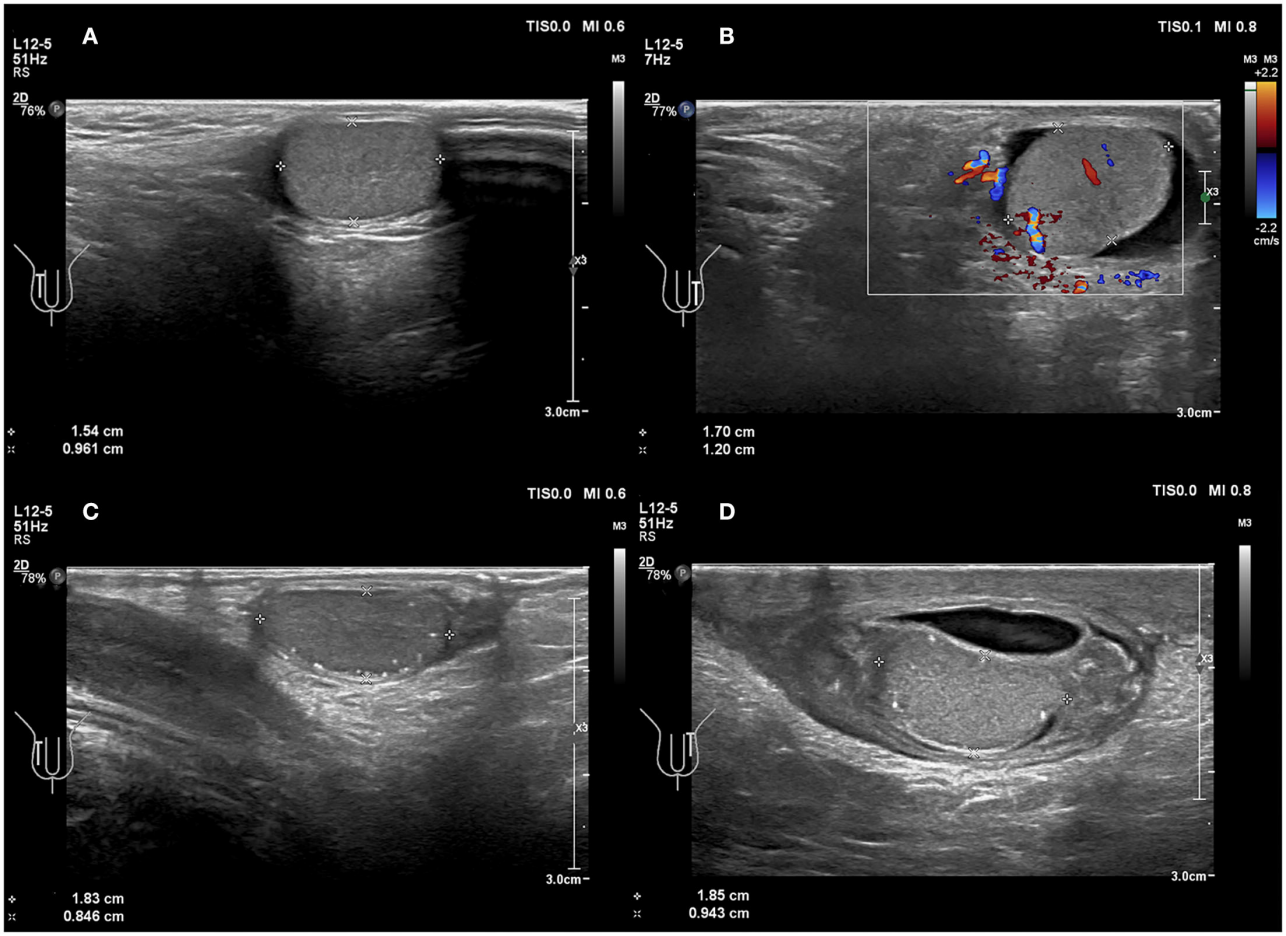
**(A,B)** A 6 years 4-month-old boy with a first episode of acute left scrotal pain for 2 days. **(A)** Normal testicle, no change in testicular axial direction. **(B)** Torsion of the testicle and the testicle change in axial direction. **(C,D)** An 8 years 3-month-old boy with the third episode of acute left scrotal pain for 5 days; initially misdiagnosed as epididymitis. **(C)** Normal testicle, no change in testicular axial direction. **(D)** Torsion of the testicle, the testicle change in axial direction.

The testes are commonly in an upright position. If the long axis of the testicle is inclined or even horizontal relative to the long axis of the thigh or long axis of the femur, especially if the orientation of the testicular mediastinum changes in the cross-section, it may indicate testicular torsion (28). In present study, four testicles (4/7 = 57.1%) were oblique (Figure 3). Previous studies have reported that testicular torsion was more likely to occur on the left side (5, 29). A study by Obi showed that the left testis was more often involved than the right: 53.3 vs. 37.8% (30) because the left spermatic cord is longer than the right. In addition, our study found that four testicular torsions (4/7 = 57.1%) occurred on the left, whereas three testicular torsions (3/7 = 42.8%) occurred on the right side, which was consistent with previous studies. However, studies with larger sample sizes are warranted.

### Heterogeneous echotexture of the testicles

**Figure 4:**
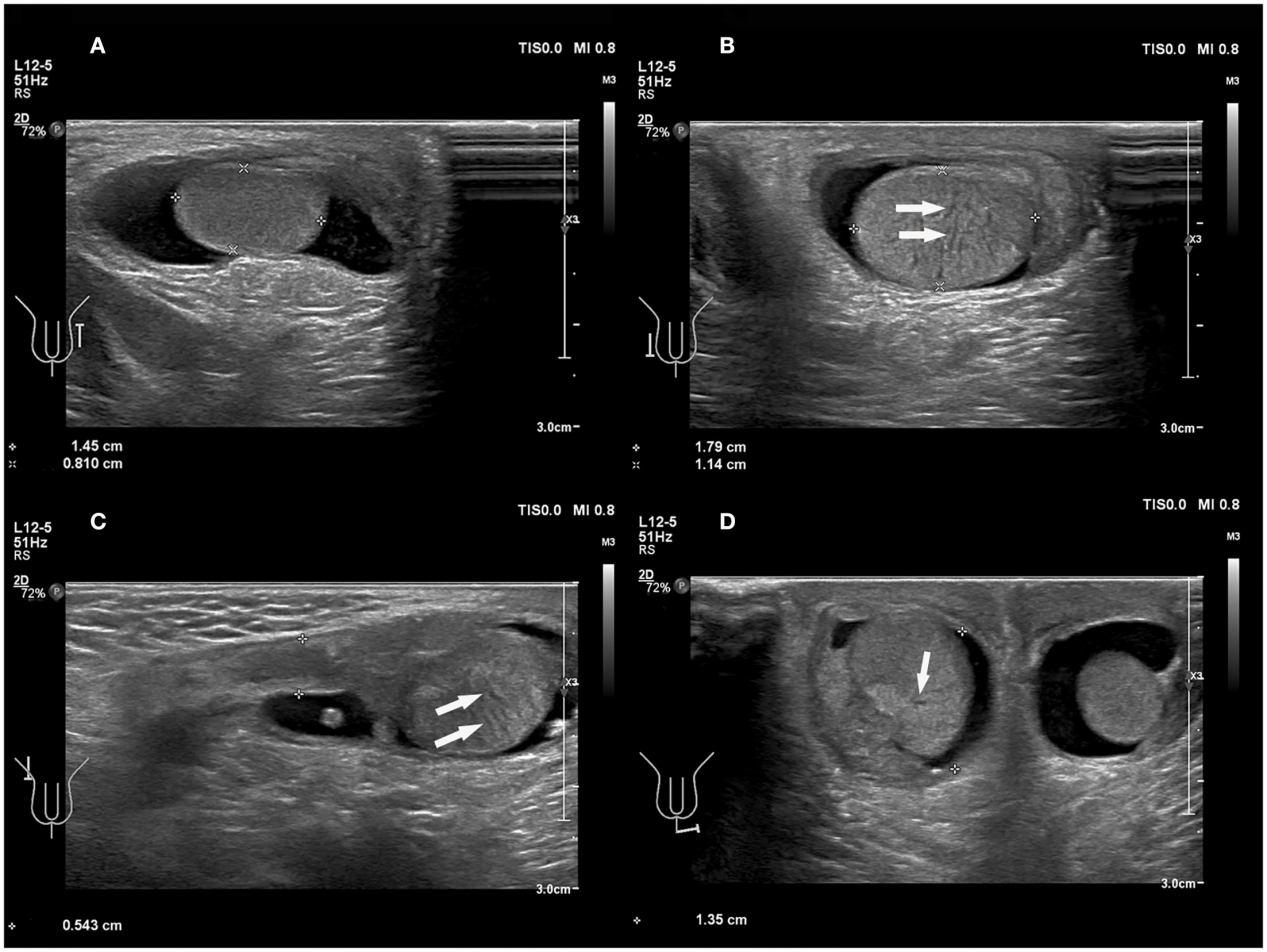
A 49 days old boy with a first episode of acute right scrotal swelling for 4 days. **(A)** Normal testicle, in which echo was even. **(B–D)** Torsion of the testicle; the echo of testicular parenchyma was uneven and anechoic fissure arounded the mediastinum (arrows).

Testicular torsion can be present with various degrees of twist, thus resulting in the heterogeneous parenchymal echotexture seen in testicular torsion. Assessing echogenicity is useful in predicting the likelihood of testicular salvage on scrotal exploration. The parenchymal echotexture of the affected testis, compared with the contralateral testis, has been used as an indicator for viability. Middleton et al. (31) demonstrated that sonography could provide useful information regarding testicular viability. Additionally, other studies concluded that the presence of significant heterogeneity indicated a late torsion and testicular non-viability; whereas a homogeneous signal of testicular parenchyma may indicate the viability of the testicles (32, 33). Kaye et al. (33) reported in a study involving 55 patients that heterogeneous echotexture was predictive of organ loss with an accuracy rate of 96.4%. In the present study, there were five cases (5/7 = 71.4%) with the heterogeneous echo of testis parenchyma, and anechoic fissure arounded the mediastinum (Figure 4). These results matched surgical findings. Heterogeneous echo indicated that the testicular torsion's rate was 71.4% (5/7) in the present study. So, careful mapping of testicular echotexture can help with the early diagnosis of potential segmental necrosis, and can also aid follow-up examinations when the echotexture returns to normal.

### Epididymal enlargement

Observation of the epididymis is also essential. Galina et al. (34) showed that the position and morphology of the epididymal head were abnormal in all nine of the children in that study with acute testicular torsion but preserved testicular flow. In the present study, there were enlarged epididymis heads and increased blood flow in 6 boys (6/7 = 85.7%). That is an abnormal configuration and displaced position of the head of the epididymis, which may be sonographic findings indicative of torsion. Therefore, observation of the epididymis is especially important in cases of incomplete torsion with the preserved testicular flow.

**Figure 5:**
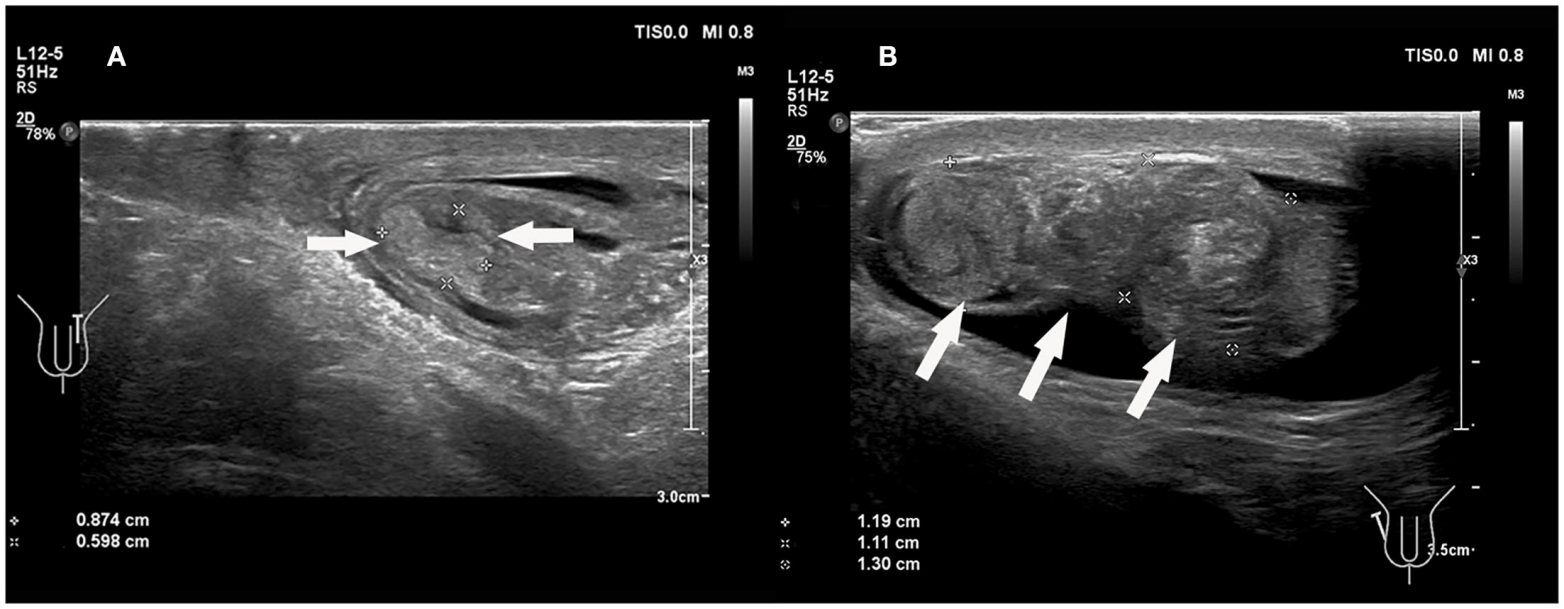
**(A)** An 8 years 3-month-old boy with the third episode of acute left scrotal pain for 5 days, initially misdiagnosed as epididymitis. **(B)** A 14 years, 3-month-old boy with a first episode of acute right scrotal pain for 7 h. The conglomerate of edematous epididymis and convoluted spermatic cord (“epididymal-cord complex”), formed pseudomass (arrows).

The epididymis is inevitably involved in torsion, and it is often enlarged. In our study, the epididymides were enlarged to varying degrees, which may be commonly misdiagnosed as epididymitis. An 8-year 3-month-old boy from the current study with a third episode of acute left scrotal pain for 5 days was initially misdiagnosed as epididymitis. In addition, the enlarged epididymis and convoluted spermatic cord are easily entangled together to form pseudo masses (Figure 5, Supplementary Video 1). The appearance of the pseudo masses was similar to that of an inflamed epididymis. A sonographic review of the spermatic cord can differentiate testicular torsion with pseudo-mass formation from epididymitis. The presence of a straight spermatic cord with a hyperemic epididymis, but no pseudo mass can be present with both epididymitis and torsion of an appendage, both of which should be treated therapeutically, not surgically (18).

### The spermatic cord–“whirlpool sign”

**Figure 6:**
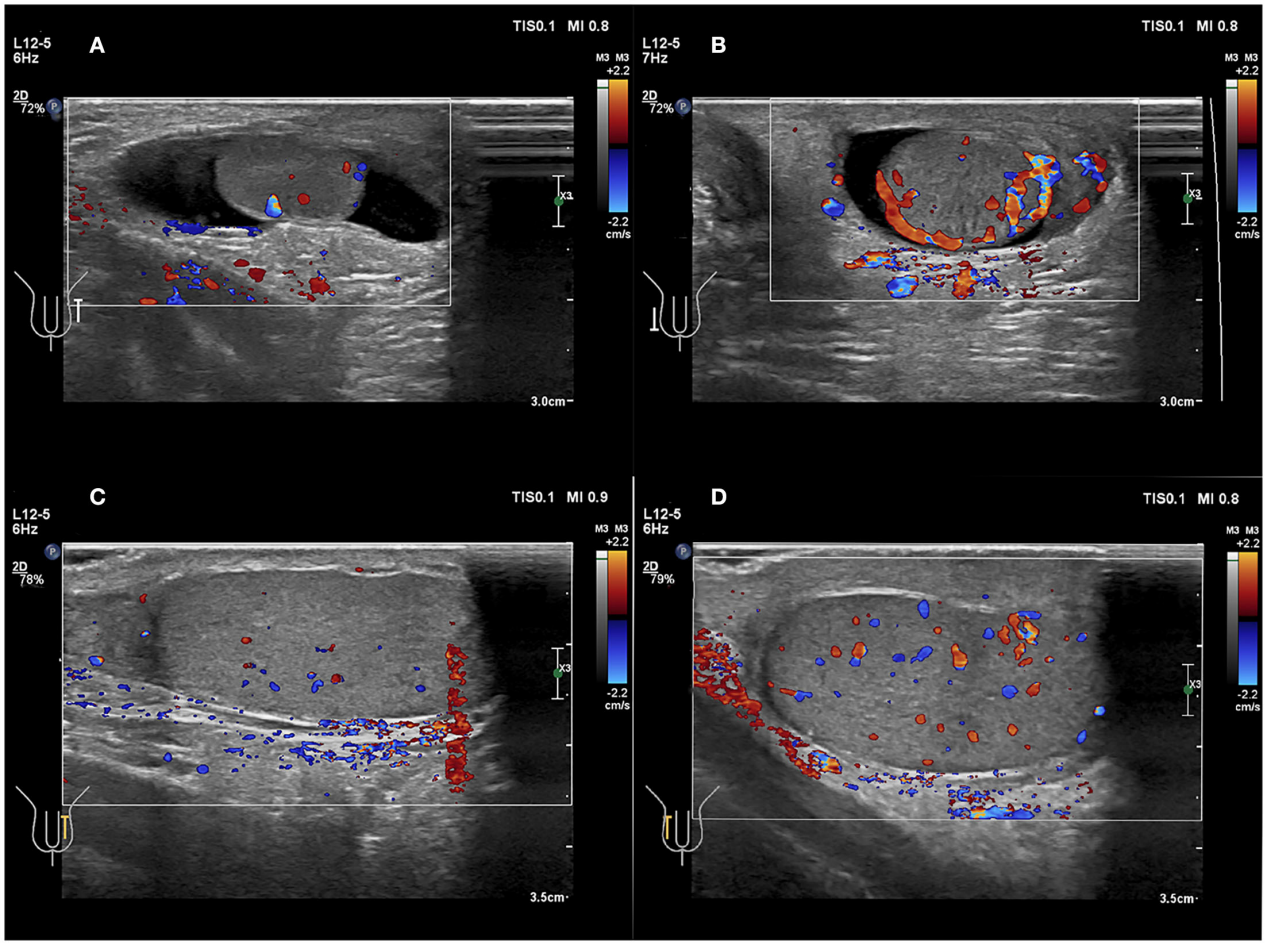
**(A,B)** A 49 days old boy with a first episode of acute right scrotal swelling for 4 days. **(C,D)** A 14 years 3-month-old boy with a first episode of acute right scrotal swelling for 7 h. **(A,C)** Normal testicle, Color Doppler showed blood flow of the testicle was normal. **(B,D)** Testicular torsion, testicular Color Doppler showed blood flow of the testicle was slightly increased.

The diagnosis of torsion is based on a lack of blood flow in the testes or a marked reduction in blood flow in the affected testes (35). In the present study, three cases (3/7 = 42.8%) showed an increased blood flow, and four cases (4/7 = 57.1%) had preserved blood flow (Figure 6); which indicated the low yield of blood perfusion in diagnosing testicular torsion by US. Four cases (4/7 = 57.1%) with hyperperfusion were described as follows: (1) Enlarged testicular volume may increase the diagnostic sensitivity of Color Doppler US (36). (2) Inflammatory response in the ischemic testicular parenchyma may increase parenchymal perfusion (37). (3) There are different types of testicular torsion, such as complete testicular torsion, intermittent testicular torsion, and partial or incomplete torsion, based on the various degrees of twist (18).

**Figure 7:**
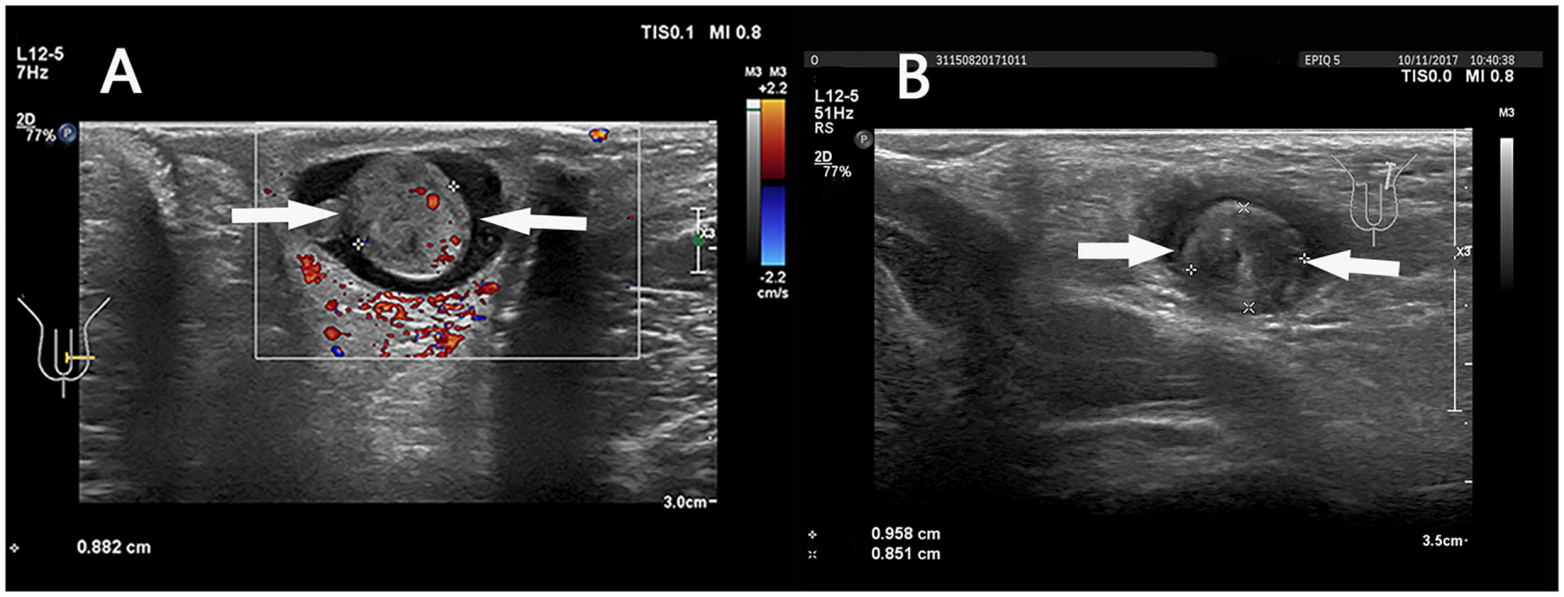
**(A)** A 6 years 4-month-old boy with a first episode of acute left scrotal pain for 2 days. **(B)** An 8 years 5-month-old boy with a first episode of acute left scrotal pain for 15 h. **(A,B)** The “whirlpool sign” in spermatic cord (arrows).

In the present study, all patients represented cases of intrathecal testicular torsion, which was confirmed by surgery, with various twisting states from 90 to 540 degrees. Taken together, the residual blood flow in all cases may be caused by intermittent or permanent spermatic cord torsion. In this case, the “whirlpool sign” was the crucial feature (Figure 7), which was consistent with early studies (6, 18, 28, 38). The “whirlpool sign” is defined as an abrupt change in the course of the spermatic cord with a spiral twist at the external inguinal ring or in the scrotal sac (26). The “whirlpool sign” of the spermatic cord is considered to be a more direct and specific sign of testicular torsion, and is valuable in the diagnosis of complete, intermittent, and incomplete torsion of the testis, regardless of Color Doppler US findings (6, 28, 38).

A meta-analysis (39) showed that the “whirlpool sign” was positive in 120 out of 184 (65.2%) patients with testicular torsion, with a pooled sensitivity of 0.73 and a pooled specificity of 0.99. This review proved that the “whirlpool sign” was present in most cases of torsion, but not in all patients. Studies in the perinatal age group did not record the presence of the “whirlpool sign” in any of the subjects with testicular torsion (40). It indicated that the value of the “whirlpool sign” in the neonatal is limited, due to anatomical differences, such as the smaller size of anatomical structures and extravaginal location.

With regard to the value of operator experience, the sensitivity for visualizing the spermatic cord twist with high-resolution ultrasonography was 81.8% in junior radiologists compared with 97.3% in senior radiologists (39). The “whirlpool sign” is a valuable characteristic in diagnosing testicular torsion. It is reliant on the operator's skill and experience. The “whirlpool sign” should not be utilized in isolation, but must be interpreted in combination with clinical findings as well as other ultrasonic features that have been described.

In patients with preserved blood flow, the position of the spermatic cord and other ultrasonic features should be assessed comprehensively, especially evaluate the full length of the spermatic cord.

### Follow-up

During the follow-up, 2 patients (2/7 = 28.5%) were lost. In addition, three patients (3/7 = 42.8%) underwent testicular ultrasound within 1 month after operation, 1 patient (1/7=14.3%) within 3 months, and 1 patient (1/7 = 14.3%) within 6 months, and ultrasound images indicated there were no complications in these testes at the operating side.

### Study limitations

This study had some limitations. First, the study has a small sample size. Second, all patients were tested by Color Doppler; however, resistive index (RI) and venous flow were not assessed. Third, we did not recruit normal volunteers as controls, but instead, used the opposite side of the healthy testes as the reference group. Fourth, the quality of a sonogram is dependent on the child's condition and the sonographer's skill, and these factors may have affected the results of this study. In future, prospective, double blind, case-control studies with larger sample sizes should be conducted to confirm our findings.

## Conclusion

In our retrospective study, 7 cases of testes with preserved flow, we can not diagnose testicular torsion based on Color Doppler. The results showed that testicular torsion was confirmed in all 7 patients after surgery. This gives us a great indicator, when we examine pediatrics with testicular swelling and pain, we should not give a diagnosis of testicular torsion only based on blood flow signals. Testes with Color Doppler flow signals might be obstacles to surgical exploration, with resultant testicular loss in false-negative cases. We should further observe the morphology and position of the testes and epididymis, the echo of the testicular parenchyma, and, especially evaluate the spermatic cord throughout its course, whether there is a “whirlpool sign” or not, so as to avoid missing testicular torsion with preserved flow.

## Data availability statement

The original contributions presented in the study are included in the article/Supplementary material, further inquiries can be directed to the corresponding author.

## Ethics statement

The studies involving human participants were reviewed and approved by Research Ethics Committee of The Second Affiliated Hospital of Wenzhou Medical University. Written informed consent from the participants' legal guardian/next of kin was not required to participate in this study in accordance with the National Legislation and the Institutional Requirements.

## Author contributions

Conception and design: ZX and HL. Administrative support: HL. Provision of study materials or patients: SN and JW. Collection and assembly of data: ZX and JW. Data analysis and interpretation: ZX and SN. Manuscript writing and final approval of manuscript: All authors.

## Conflict of interest

The authors declare that the research was conducted in the absence of any commercial or financial relationships that could be construed as a potential conflict of interest.

## Publisher's note

All claims expressed in this article are solely those of the authors and do not necessarily represent those of their affiliated organizations, or those of the publisher, the editors and the reviewers. Any product that may be evaluated in this article, or claim that may be made by its manufacturer, is not guaranteed or endorsed by the publisher.

## Supplementary material

Supplementary Video 1 The conglomerate of edematous epididymis and convoluted spermatic cord—“epididymal-cord complex” to form pseudomass.

## Abbreviations

US: Ultrasonography
PACS: Picture Archiving and Communication System.

## References

[1] Sung EK Setty BN Castro-Aragon I. Sonography of the pediatric scrotum: emphasis on the Ts–torsion, trauma, and tumors. AJR. (2012) 198:996–1003. 10.2214/AJR.11.8034 22528888

[2] Boettcher M Bergholz R Krebs TF Wenke K Aronson DC. Clinical predictors of testicular torsion in children. Urology. (2012) 79:670–4. 10.1016/j.urology.2011.10.041 22386422

[3] Yang C Song B Tan J Liu X Wei GH. Testicular torsion in children: a 20-year retrospective study in a single institution. Scientific World J. (2011) 11:362–8. 10.1100/tsw.2011.39 21336452 PMC5720097

[4] Pogorelić Z Mustapić K Jukić M Todorić J Mrklić I Mešštrović J. Management of acute scrotum in children: a 25-year single center experience on 558 pediatric patients. Can J Urol. (2016) 23:8594–601. 27995859

[5] Williamson RC. Torsion of the testis and allied conditions. Br J Surg. (1976) 63:465–76. 10.1002/bjs.1800630618 6106

[6] Vijayaraghavan SB. Sonographic differential diagnosis of acute scrotum: real-time whirlpool sign, a key sign of torsion. J Ultrasound Med. (2006) 25:563–74. 10.7863/jum.2006.25.5.563 16632779

[7] Janetschek G Schreckenberg F Mikuz G Marberger M. Experimental testicular torsion: effect on endocrine and exocrine function and contralateral testicular histology. Urol Res. (1988) 16:43–7. 10.1007/BF00264627 3125648

[8] Howe AS Vasudevan V Kongnyuy M Rychik K Thomas LA Matuskova M. Degree of twisting and duration of symptoms are prognostic factors of testis salvage during episodes of testicular torsion. Transl Androl Urol. (2017) 6:1159–66. 10.21037/tau.2017.09.10 29354505 PMC5760391

[9] Davenport M. ABC of general surgery in children. Acute problems of the scrotum. BMJ. (1996) 312:435–7. 10.1136/bmj.312.7028.435 8601119 PMC2350084

[10] Jefferson RH Perez LM Joseph DB. Critical analysis of the clinical presentation of acute scrotum: a 9-year experience at a single institution. J Urol. (1997) 158:1198–200. 10.1016/S0022-5347(01)64426-4 9258172

[11] Patriquin HB Yazbeck S Trinh B Jequier S Burns PN Grignon A. Testicular torsion in infants and children: diagnosis with Doppler sonography. Radiology. (1993) 188:781–5. 10.1148/radiology.188.3.8351347 8351347

[12] Burks DD Markey BJ Burkhard TK Balsara ZN Haluszka MM Canning DA. Suspected testicular torsion and ischemia: evaluation with color Doppler sonography. Radiology. (1990) 175:815–21. 10.1148/radiology.175.3.2188301 2188301

[13] Gunther P Schenk JP Wunsch R Holland-Cunz S Kessler U Troger J. Acute testicular torsion in children: the role of sonography in the diagnostic workup. Eur Radiol. (2006) 16:2527–32. 10.1007/s00330-006-0287-1 16724203

[14] Waldert M Klatte T Schmidbauer J Remzi M Lackner J Marberger M. Color Doppler sonography reliably identifies testicular torsion in boys. Urology. (2010) 75:1170–4. 10.1016/j.urology.2009.07.1298 19913882

[15] Kravchick S Cytron S Leibovici O Linov L London D Altshuler A. Color Doppler sonography: its real role in the evaluation of children with highly suspected testicular torsion. Eur Radiol. (2001) 11:1000–5. 10.1007/s003300000695 11419144

[16] Dogra VS Rubens DJ Gottlieb RH Bhatt S. Torsion and beyond: new twists in spectral Doppler evaluation of the scrotum. J Ultrasound Med. (2004) 23:1077–85. 10.7863/jum.2004.23.8.1077 15284466

[17] Yusuf GT Sidhu PS. A review of ultrasound imaging in scrotal emergencies. J Ultrasound. (2013) 16:171–8. 10.1007/s40477-013-0033-x 24432171 PMC3846954

[18] Munden MM Williams JL Zhang W Crowe JE Munden RF Cisek LJ. Intermittent testicular torsion in the pediatric patient: sonographic indicators of a difficult diagnosis. AJR. (2013) 201:912–8. 10.2214/AJR.12.9448 24059384

[19] Prando D. Torsion of the spermatic cord: the main gray-scale and doppler sonographic signs. Abdom Imaging. (2009) 34:648–61. 10.1007/s00261-008-9449-8 18709404

[20] AIUM Practice Guideline for the Performance of Scrotal Ultrasound Examinations. J Ultrasound Med. (2015) 34:1–5. 10.7863/ultra.34.8.15.13.0006 26206818

[21] Yidong L Guo LX. Diagnosis and treatment of testicular torsion safety consensus. J Modern Depart Urol. (2019) 24:434–7. 10.3969/j.issn.10098291.2019.06.004

[22] Osumah TS Jimbo M Granberg CF Gargollo PC. Frontiers in pediatric testicular torsion: an integrated review of prevailing trends and management outcomes. J Pediatr Urol. (2018) 14:394–401. 10.1016/j.jpurol.2018.07.002 30087037

[23] Monteilh C Calixte R Burjonrappa S. Controversies in the management of neonatal testicular torsion: a meta-analysis. J Pediatr Surg. (2019) 54:815–9. 10.1016/j.jpedsurg.2018.07.006 30098810

[24] Velasquez J Boniface MP Mohseni M. Acute Scrotum Pain. StatPearls. Treasure Island (FL): StatPearls Publishing Copyright © 2022. StatPearls Publishing LLC. (2022).

[25] Mellick LB Sinex JE Gibson RW Mears K. A systematic review of testicle survival time after a torsion event. Pediatr Emerg Care. (2019) 35:821–5. 10.1097/PEC.0000000000001287 28953100

[26] Bandarkar AN Blask AR. Testicular torsion with preserved flow: key sonographic features and value-added approach to diagnosis. Pediatr Radiol. (2018) 48:735–44. 10.1007/s00247-018-4093-0 29468365 PMC5895684

[27] Boettcher M Krebs T Bergholz R Wenke K Aronson D Reinshagen K. Clinical and sonographic features predict testicular torsion in children: a prospective study. BJU Int. (2013) 112:1201–6. 10.1111/bju.12229 23826981

[28] Baud C Veyrac C Couture A Ferran JL. Spiral twist of the spermatic cord: a reliable sign of testicular torsion. Pediatr Radiol. (1998) 28:950–4. 10.1007/s002470050507 9880639

[29] Skoglund RW McRoberts JW Ragde H. Torsion of the spermatic cord: a review of the literature and an analysis of 70 new cases. J Urol. (1970) 104:604–7. 10.1016/S0022-5347(17)61792-0 5476477

[30] Obi AO. Intermittent testicular torsion. Niger J Clin Pract. (2017) 20:1273–6. 10.4103/njcp.njcp_218_16 29192631

[31] Middleton WD Middleton MA Dierks M Keetch D Dierks S. Sonographic prediction of viability in testicular torsion: preliminary observations. J Ultrasound Med. (1997) 16:23–7. 10.7863/jum.1997.16.1.23 8979223

[32] Eaton SH Cendron MA Estrada CR Bauer SB Borer JG Cilento BG. Intermittent testicular torsion: diagnostic features and management outcomes. J Urol. (2005) 174:1532–5. 10.1097/01.ju.0000177726.84913.cc 16148646

[33] Kaye JD Shapiro EY Levitt SB Friedman SC Gitlin J Freyle J. Parenchymal echo texture predicts testicular salvage after torsion: potential impact on the need for emergent exploration. J Urol. (2008) 180:1733–6. 10.1016/j.juro.2008.03.104 18721947

[34] Galina P Dermentzoglou V Baltogiannis N Zarifi M. Sonographic appearances of the epididymis in boys with acute testicular torsion but preserved testicular blood flow on color Doppler. Pediatr Radiol. (2015) 45:1661–71. 10.1007/s00247-015-3375-z 26104655

[35] Hormann M Balassy C Philipp MO Pumberger W. Imaging of the scrotum in children. Eur Radiol. (2004) 14:974–83. 10.1007/s00330-004-2248-x 14986053

[36] Ingram S Hollman AS. Color Doppler sonography of the normal pediatric testis. Clin Radiol. (1994) 49:266–7. 10.1016/S0009-9260(05)81854-9 8162685

[37] D'Andrea A Coppolino F Cesarano E Russo A Cappabianca S Genovese EA. US in the assessment of acute scrotum. Crit Ultrasound J. (2013) 5 Suppl 1:S8. 10.1186/2036-7902-5-S1-S8 23902859 PMC3711727

[38] Cassar S Bhatt S Paltiel HJ Dogra VS. Role of spectral Doppler sonography in the evaluation of partial testicular torsion. J Ultrasound Med. (2008) 27:1629–38. 10.7863/jum.2008.27.11.1629 18946103

[39] McDowall J Adam A Gerber L Enyuma COA Aigbodion SJ Buchanan S. The ultrasonographic “whirlpool sign” in testicular torsion: valuable tool or waste of valuable time? A systematic review and meta-analysis. Emergency Radiol. (2018) 25:281–92. 10.1007/s10140-018-1579-x 29335899

[40] Xiao H Gao Y Li Y Tang Y Zhu L Xu J. Ultrasound assessment of perinatal testicular torsion. Br J Radiol. (2016) 89:20151077. 10.1259/bjr.20151077 27278088 PMC5124879

